# Trap of weights: The reuse of weights in the floating catchment area (FCA) methods to measuring accessibility

**DOI:** 10.12688/f1000research.51483.2

**Published:** 2022-02-08

**Authors:** Lina Zhang

**Affiliations:** 1 Department of Urban and Regional Planning, The Faculty of Spatial Planning, TU Dortmund University, Dortmund, 44149, Germany

**Keywords:** Spatial accessibility, geographic weights, E2SFCA, M2SFCA, 3SFCA

## Abstract

**Background:** Geographic weights are vital in the floating catchment area (FCA) method of accessibility measurements due to their simulation of spatial barriers in various ways. When modelling population demand, geographical weights with different distance decay coefficients can reflect diverse distance tolerances in facility utilization and could lead to erratic accessibility results. Quantifying accessibility as the sum of weighted supply-demand ratios can alleviate the distance decay coefficient's influence and generate stable geographic patterns. However, the effects of weighted ratios on different FCA models and resources have not been investigated.

**Methods:** To identify impacts of weighted ratios on various FCA variants, this study contrasted the accessibility calculated from the sum of ratios (access) and the sum of weighted ratios (access ratios) within three prevalent FCA models: enhanced two-step FCA (E2SFCA), modified two-step FCA (M2SFCA), and three-step FCA (3SFCA). In addition, the accessibilities of various resources evaluate the stability of the weighted ratios' effect. This study therefore examined the accessibilities to primary schools, job opportunities, and major hospitals in Shanghai. Shanghai is a case study that provides lessons on using big data to measure accessibility in metropolitan areas.

**Results:** Geographic weights can not only mitigate the impact of the distance decay coefficients, but can also eliminate model features, which reduces the performance of the M2SFCA's supply decay and the 3SFCA's population demand adjustment in accessibility results. Moreover, weighted ratios tend to overestimate accessibility in marginal communities that lie within fewer catchments, regardless of the resource type. This tendency can lead to an epistemological trap that creates an inaccurate and counter-intuitive perception of resource distribution in a given area.

**Conclusions:** The results identify a gap between the methodological logic and the empirical perception in accessibility measurements. This study concludes that the use of geographic weights needs to be cautious and epistemologically consistent.

## Introduction

Accessibility is a fundamental indicator for detecting spatial equity in distributive justice ( Talen, 2001). Derived from Rawls’s philosophy of justice, distributive justice offers deontological guidance on distributions of benefits and burdens across members of the society ( Lamont, 2017; Rawls, 1971). Distributive justice is a classic and basic theme ( Arrow, 1973), as distributions of benefits and burdens have a substantial impact on people’s lives and social inequalities. In essence, distributive justice includes guidance for political processes and structures, while spatial equity more specifically examines the social impact of the physical distribution of certain goods and services ( Dadashpoor et al., 2016; Taleai et al., 2014). Accessibility describes the distributive supply-demand relationship of resources and can be visualised using various geographic information system (GIS) models ( Higgs, 2004; Luo and Wang, 2003). Accurate measurements of accessibility are crucial for identifying areas of poor resource distribution ( Yang et al., 2006), revealing spatial reflections of social inequality ( Guagliardo, 2004), and providing policymakers with practical suggestions on how to achieve distributive justice ( Neutens, 2015).

It is important to consider methodologies in accessibility measurements ( Baradaran and Ramjerdi, 2001; Bell et al., 2013; Sherman et al., 2005). On one hand, the use of different models, which yield divergent outcomes, indicates that there are heterogeneous understandings of accessibility ( Bunel and Tovar, 2013; Guagliardo, 2004). For example, the buffer model ( Nicholls, 2001) defines accessibility in terms of facilities’ service areas, which are measured by the radial distance from a given facility, while the isochronic model ( Cervero et al., 1998) uses the travel time to the facilities as the main indicator of accessibility. The gravity model originally defined accessibility within a census tract as the number of facilities divided by the distance between the facilities and the census tract ( Hansen, 1959). Weibull (1976) later added population demand to the denominator to simulate the competition among residents for limited capacity services. On other hand, manifold factors in GIS models can affect the distributive pattern of accessibility. These factors include spatial scale ( Higgs, 2004), transport model ( Mao and Nekorchuk, 2013; Ni et al., 2019), unit aggregation ( Apparicio et al., 2008; Hewko et al., 2002), and distance decay function ( Kwan, 1998).

Floating catchment area (FCA) metrics have been among the most widely applied models in accessibility measurements ( Neutens, 2015). Based on service area, FCA metrics utilize the catchment concept to describe the efficacy area of a location, including the possible geographical area that describes resident activities for a given community. In FCA methods, accessibility within a census tract is defined as the sum of the facilities’ supply-demand ratios within its catchment ( Luo and Wang, 2003). A strength of FCA metrics is that it combines the advantages of the gravity model and the cumulative-opportunity model. The gravity model provides a global distribution of accessibility by considering supply and consumer consumption in each area ( Geertman and Ritsema Van Eck, 1995), whereas the cumulative-opportunity model measures spatial equity in terms of potential opportunity. This combination drives the flexibility of FCA methods. Scholars have endeavoured to create various FCA variants for accurate simulation of accessibility, including the enhanced two-step FCA (E2SFCA) ( Luo and Qi, 2009), the modified two-step FCA (M2SFCA) ( Delamater, 2013), the three-step FCA (3SFCA) ( Wan et al., 2012b), and the flow-based 2SFCA method ( Tang et al., 2017).

Distance weights play an important role in FCA metrics development. The E2SFCA method applies distance weights to simulate people’s declining intent to facility utilization as the distance to them increases. Similarly, the M2SFCA considers the decrease in service capacity with increasing distance, by attaching weights in supply calculation. The 3SFCA calculates the share of distance weights in each facility to simulate the competition among suppliers. However, when applying weight formulas to represent the population demand, the value of the decay coefficient can significantly alter accessibility distribution ( Giles-Cortia and Donovan, 2002). This is because the coefficient can sensitively change the population demand and accessibility is calculated as the aggregation of supply-demand ratios ( Bauer and Groneberg, 2016). To reduce the sensitivity of the decay coefficient, Wan et al. (2012a) argue that calculating the ratio between a census tract’s accessibility and the mean accessibility of all census tract can generate a relatively stable accessibility result. This repeated use of distance weights in both first and second steps of 2SFCA methods, so-called ‘the reuse of weights’, simulates spatial barriers in service capacity, population demand, and supply-demand ratios; thus, it has become a common component in FCA methods.

However, taking accessibility as the sum of weighted ratios and examining its impact on various FCA models has not yet been performed. This study focuses on the application of weights in the final step of FCA methods. In the second step of original 2SFCA method, accessibility of certain census tract is the sum of supply-demand ratios of the facilities lying the catchment of the census tract ( Luo and Wang, 2003). Later, Luo and Qi (2009) improved the second step in the E2SFCA method, added distance weights and multiplied it by the original supply-demand ratios. Therefore, accessibility of certain census tract becomes the sum of weighted ratios, which firstly multiplies the facilities' supply-demand ratios by distance weights, and then adds those weighted ratios up. This improved method transforms the accessibility from a sum of opportunities to an accumulation of weighted ones.

For clarity, this study only focuses on the application of weights in the final step of the FCA methods; defines the original non-weighted accessibility as ‘access’ and the weighted ratios as ‘access ratios’. Applying this method also raises the question of the extent to which weighted ratios provide stability in different FCA variants. Wan et al. (2012a) demonstrate how weighted ratios provide resistance to varying values of the distance decay coefficient in the E2SFCA, while this conceptual transformation could lead to stability across FCA models and potential fallacies. To systematically measure the impact of reusing weights, this study uses three prevalent FCA models (i.e. E2SFCA, M2SFCA, and 3SFCA) to compare accessibility as the sum of *ratios* and accessibility as the sum of *weighted ratios.* In addition, this research uses Shanghai as a case study to provide accessibility measurements based on big data and metropolitan case evidence. Shanghai is one of the most populated metropolises in the People’s Republic of China, with a population of 24.20 million in 2017 ( Shanghai Statistics Bureau, 2018). During its rapid urbanisation, social inequality has been a severe problem ( Li and Wu, 2008), and accessibility measurements are necessary for its distributive justice and sustainable development. Based on point of interest (POI) data from 2017, this study compares the accessibility of three resources in Shanghai, namely primary schools, job opportunities, and major hospitals. By comparing the impact of reusing weights on different FCA models and resources, the study identifies the trap of weights, provides empirical evidence for the gap between methodology and epistemology, and justifies the importance of epistemological criteria in the evaluation of accessibility methods.

## Reusing weights in accessibility measurements

### Evaluation of weights under the complexity of accessibility measurements

The complexity in modelling spatial accessibility is derived from its abundant definitions and methodological flexibility, which vary across different fields and build distinct taxonomies. For example, in transportation, accessibility is defined as the potential to reach spatially dispersed opportunities, with a focus on travel cost and transport policy ( Páez et al., 2012). In health studies, accessibility of medical facilities is one of the five dimensions (accessibility, availability, accommodation, affordability, and acceptability) that describe the relationship between health facilities and patient utilization ( Penchansky and Thomas, 1981). Guagliardo (2004) deconstructs the concept of health accessibility into two stages (potential and realized) and two dimensions (spatial and aspatial). The former refers to the difference between potential opportunity simulation and actual utilization, while the latter considers spatial factors and socioeconomic attributes.

The many definitions of ‘accessibility’ lead to a variety of accessibility-modelling methodologies. The initial container method takes accessibility as the number of facilities in a given unit ( Talen and Anselin, 1998). The minimum distance and travel cost methods prioritise transportation in determining facility utilization ( Guy, 1983). The Kernel density method estimates the relationship between facility density and population density ( Yang et al., 2006). However, few models consider user behaviour in their measurements. For example, residents’ facility utilization can extend beyond their communities, and has a declining frequency as travel distance increases ( Higgs, 2004). This is due to so-called ‘spatial barriers’ ( Guagliardo et al., 2004; Neutens, 2015). To account for this, the gravity model introduces a distance decay function in assessing population demand and defines accessibility as the sum of facilities’ supply-demand ratios related to a given community. FCA methods are special applications of the gravity model that use catchment areas to simulate the geographical sphere of facility utilization and calculate the supply-demand ratios within catchments ( Luo and Wang, 2003).

Numerous factors in GIS models affect the distributive patterns of accessibility maps. Different unit aggregations can generate divergent distributions because of the modifiable areal unit problem (MAUP) ( Bell et al., 2013; Dadashpoor et al., 2016). As a long-standing statistical bias, the MAUP refers to the fact that different shapes and sizes of the unit can alter the geographic results ( Hewko et al., 2002; Zhang et al., 2011). Bell et al. (2013) contrasted accessibility in neighbourhood and census tract to highlight the social characteristic of the unit aggregation. Additional confounding includes the methods of calculating distance, transport mode, catchment size, and facility capacity ( Ni et al., 2019; Pan et al., 2018; Wan et al., 2012b). Apparicio et al. (2008) conducted a comprehensive comparison to examine the influence of different distance types and units aggregation. Geographic weights also play an important role in the accessibility measurements, as illustrated in the next section.

As this study aims to examine the influence of reusing weights in the last step of the FCA methods, the complexity of modelling accessibility increases the difficulty of selecting appropriate evaluation dimensions for their impact. Despite the mentioned influencers, the study chose two dimensions—model type and resources—as the evaluation dimensions for the impact of the reusing weights. Bunel and Tovar (2013) identified the model type as a key issue that can generate different empirical results in accessibility measurements. Although the methodological improvements have driven the reuse of weights, it is a conceptual transform in the FCA methods ( Wan et al., 2012a). This conceptual shift should be investigated in a variety of FCA variants for its legitimacy. Moreover, the FCA methods are applied in measuring various resources’ accessibility, including health facilities, jobs, and urban parks ( Delamater et al., 2019; Kawabata and Takahashi, 2005; Xie et al., 2018). The wide application of the FCA methods to different resources reflects the fact that the rationality of the methodology can transcend the resource characteristics. This means that differences in resource types do not affect the validity of the method. Thus, comparing the same method across resources allows further testing of the methodological generality. This study introduces a dimension of model type to test the legitimacy of reusing weights and a dimension of resources to examine its generality. The following section reviews how methodological improvements have driven the reuse of weights in FCA methods, as well as the potential limitations of reusing weights.

### The development of FCA methods: The ‘omnipotent’ weights

Distance weights play a major role in the development of FCA methods ( Langford et al., 2012). A series of methodological improvements have focused on changes in the application of weights for varied purposes ( Luo and Qi, 2009; Wan et al., 2012a, b). The original 2SFCA ( Luo and Wang, 2003) has two calculation stages: first, the population demand within the supplier’s catchment area is summed to calculate the supply-demand ratio for each supplier. Second, these supply-demand ratios are then summed within the catchment based on the neighbour tracts, which is the value of access (see Equation (1)).

$$A_{i}^{2S}=\underset{j\in C_{i}}{\sum }R_{j}=\underset{j\in C_{i}}{\sum }\frac{S_{j}}{\sum_{k\in C_{j}}D_{k}}$$ (1)

$A_{i}^{2S}$ represents the access at tract *i* based on the 2SFCA method, *C*_*i*_ is the catchment centred at tract *i*, *R*_*j*_ is the supply-demand ratio of supplier *j* which falls in the catchment centred at tract *i*, *S*_*j*_ is the supply capacity of supplier *j*, *C*_*j*_ is the catchment centred at supplier *j*, and *D*_*k*_ is the population demand of tract *k*, which falls in the catchment centred at supplier *j.*

The E2SFCA method enhances the stimulation of population demand in 2SFCA ( Luo and Qi, 2009). It applies a distance decay function in the population demand to change it from a dichotomous calculation to a continuous variable. Equation (2b) is the distance decay function in the form of a power function. The distance weight and population demand decline as the distance increases.

$$A_{i}^{E2}=\underset{j\in C_{i}}{\sum }R_{j}^{E2}=\underset{j\in C_{i}}{\sum }\frac{S_{j}}{\sum_{k\in C_{j}}w_{kj}\cdot D_{k}}$$ (2a)

$$w_{kj}=d_{kj}^{-\beta }$$ (2b)

$A_{i}^{E2}$ represents the access calculated by the E2SFCA method, $R_{j}^{E2}$ is the supply-demand ratio of supplier *j* which falls in the catchment centred at tract *i* calculated by the E2SFCA method, *w*_*kj*_ is the distance weight between tract *k* and supplier *j*, *d*_*kj*_ is the distance between tract *k* and supplier *j*, *β* is the decay coefficient, and the other variables are the same as in equation ( 1).

$$A_{i}^{M2}=\underset{j\in C_{i}}{\sum }R_{j}^{M2}=\underset{j\in C_{i}}{\sum }\frac{w_{ij}\cdot S_{j}}{\sum_{k\in C_{j}}w_{kj}\cdot D_{k}}$$ (3a)

$$w_{kj}=d_{kj}^{-\beta },w_{ij}=d_{ij}^{-\beta }$$ (3b)

Furthermore, the M2SFCA method argues that not only population demand decreases with distance, but also service effectiveness of suppliers ( Delamater, 2013). In M2SFCA, the effectiveness is calculated using distance weights plus the supply capacity for each pair of tracts and suppliers (see Equation (3a)). $A_{i}^{M2}$ represents the access at tract *i* based on the M2SFCA method, *w*_*ij*_ is the distance weight between tract *i* and supplier *j.* Equation (3b) is the distance decay function, *d*_*ij*_ is the distance between tract *i* and supplier *j*, and the other variables are the same as in Equation (2a) and (2b).

$$A_{i}^{3S}=\underset{j\in C_{i}}{\sum }R_{j}^{3S}=\underset{j\in C_{i}}{\sum }\frac{S_{j}}{\sum_{k\in C_{j}}w_{kj}\cdot w_{kj}^{S}\cdot D_{k}}$$ (4a)

$$w_{kj}^{S}=\frac{w_{kj}}{\sum_{m\in C_{k}}w_{km}}$$ (4b)

Unlike M2SFCA, the 3SFCA method tries to solve the exaggeration of population demand in the E2SFCA ( Wan et al., 2012b). As in Equation (2a), the population of tract *k* is calculated multiple times in accordance with the number of suppliers within tract *k*’s catchment. The solution is to introduce the supplier weights into population demand. For certain tract *k*, its supplier weight of supplier *j* ( $w_{kj}^{S}$ ) equals its distance weights *w*_*kj*_ divided by the sum of the distance weights of all suppliers within its catchment. In Equation (4a), $A_{i}^{3S}$ represents the access at tract *i* based on the 3SFCA method, *w*_*kj*_ is the distance weight between tract *k* and supplier *j.* Equation (4b) is the calculation of supplier weight $w_{kj}^{S}$ between tract *k* and supplier *j*, where supplier *m* is the supplier’s fall in the catchment of tract *k*, and *w*_*km*_ is the distance weights between tract *k* and supplier *m.*

**Figure 1.**
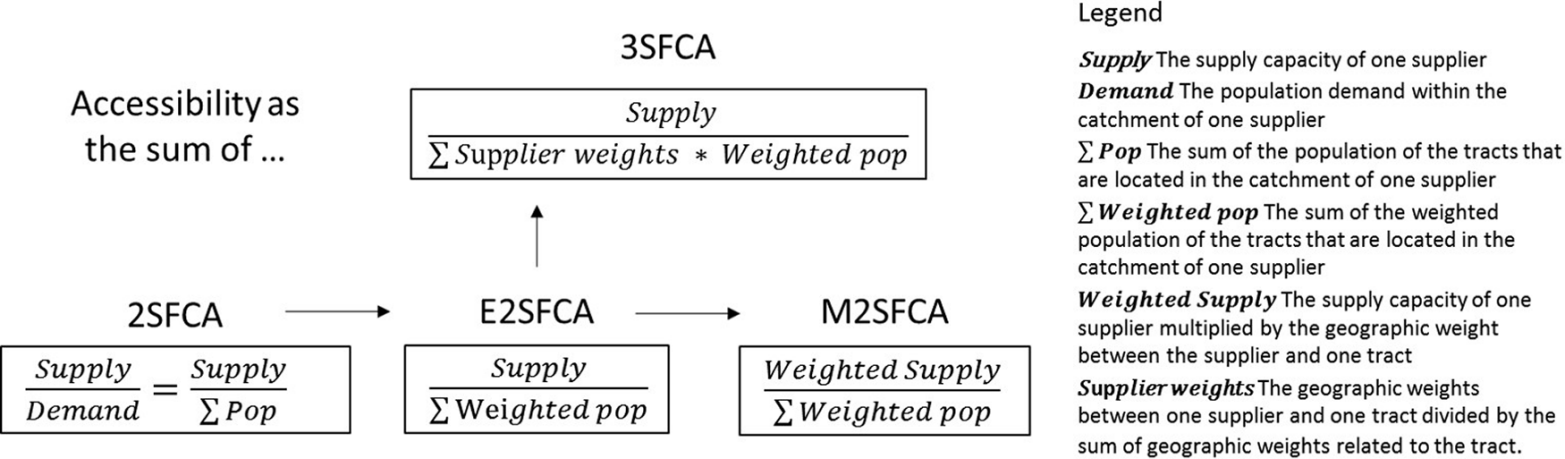
The development of floating catchment area (FCA) methods, where E2SFCA is the enhanced two-step FCA, M2SFCA is the modified two-step FCA, 3SFCA is the three-step FCA, and 2SFCA is the flow-based method. The adaptation of geographic weights has continued throughout the improvements.

Figure 1 shows the development of four FCA methods, as well as their differences. For various intents, the adaptation of geographic weights has continued throughout the FCA improvement process. Based on the original 2SFCA, the E2SFCA introduces distance weights in population demand to model spatial barriers in resource utilization, which results in declining population demand as the distance between the supplier and the consumer increases. Further to this, the M2SFCA has placed distance weights on the supply capacity to model distance barriers in resource effectiveness. The 3SFCA improves on the E2SFCA’s population demand calculation by factoring in the supplier weight. For a distance between a census unit and a supplier, its supplier weight equals its distance weight divided by the sum of the distance weights of all suppliers that are located in the catchment area of the unit. The supplier weight distributes population demand in accordance with spatial barriers and simulates the competition among suppliers. Especially in the M2SFCA and the 3SFCA, the reuse of weights is the main method to achieve their improvement goals.

Similarly, geographic weights are the solution to the high sensitivity of the distance decay coefficient, which is a common problem in the methods described above ( Xing et al., 2018). The value of the distance decay coefficient *β* (see Equation (2b), (3b), and (4b)) can significantly change the accessibility distribution, even from a monocentric to a decentralised structure. This raises two questions: how should a suitable value for the decay coefficient be determined, and to what extent should it influence the accessibility distribution? For the first question, the normal arbitrary value of *β* ranges from 1.0 to 2.2 ( Luo and Wang, 2003; Luo and Qi, 2009). One practical and rationale way to determine the coefficient values for the different facilities is to use questionnaires to simulate the spatial barriers in facility utilization ( Giles-Cortia and Donovan, 2002). For the second question, Wan et al. (2012a) argue that accessibility distribution should remain stable regardless of the coefficient value. This is because the decay coefficient describes how utilization intention and service effectiveness declines with increasing distance. For example, when *β* equals 1, the distance weight for a census tract 5 km from the hospital equals 0.2, while when *β* is 2, the weight equals 0.04 (see Equation (2b)). Both values can be appropriate according to divergent individual preferences. However, it is difficult to deal with the uncertainty around the individual use of facilities in the place-based FCA methods. Their solution is to retain the distance-decaying nature, but to remove the influence of *β* by taking accessibility as the sum of weighted supply-demand ratios, i.e. the access ratio. The distribution of access ratio is relatively stable, with similar patterns under different values of the distance decay coefficient. Table 1 shows the comparison of access ( *A*_*i*_) and access ratio ( $A_{i}^{R}$ ) of the three methods. The difference between ‘access’ and ‘access ratio’ is whether to multiply the suppliers’ supply-demand ratios ( *R*_*j*_) by distance weights ( *w*_*ij*_) in the final step. The differences between E2SFCA, M2SFCA, and 3SFCA is the way to calculate the suppliers' supply-demand ratios ( *R*_*j*_). The E2SFCA calculates *R*_*j*_ as supplier capacity divided by weighted population. The M2SFCA argues that the supplier capacity also decreases while distance increases and multiplies supplier capacity by the distance weight. The 3SFCA reveals the double-counted population demand and multiplies population demand by the distance weight and the supplier weight.

**Table 1.** Model formulas of access ( *A*_*i*_) and access ratio ( $A_{i}^{R}$ ), where E2SFCA is the enhanced two-step floating catchment area (FCA), M2SFCA is the modified two-step FCA, and 3SFCA is the three-step FCA.

Methods	Access ( *A*_*i*_)	Access ratio ( $A_{i}^{R}$ )
	Accessibility as the sum of ratios	Accessibility as the sum of weighted ratios
E2SFCA	$A_{i}=\sum_{j\in C_{i}}R_{j}^{E2}$ *	$A_{i}^{R}=\sum_{j\in C_{i}}w_{ij}\cdot R_{j}^{E2}$
M2SFCA	$A_{i}=\sum_{j\in C_{i}}R_{j}^{M2}$ **	$A_{i}^{R}=\sum_{j\in C_{i}}w_{ij}\cdot R_{j}^{M2}$
3SFCA	$A_{i}=\sum_{j\in C_{i}}R_{j}^{3S}$	$A_{i}^{R}=\sum_{j\in C_{i}}w_{ij}\cdot w_{ij}^{S}\cdot R_{j}^{3S}$ ***

* *C*_*i*_ is the catchment centred at tract *i.* $R_{j}^{E2}$ is the supply-demand ratio of supplier *j* which falls in the catchment centred at tract *i* calculated by the E2SFCA method.

** The main difference between E2SFCA, M2SFCA and 3SFCA is the way in which suppliers’ supply-demand ratios are calculated, as Figure 1 shows.

*** *w*_*ij*_ is the distance weight between tract *i* and supplier *j.* $w_{ij}^{S}$ is the supplier weight between tract *i* and supplier *j.*

**Figure 2.**
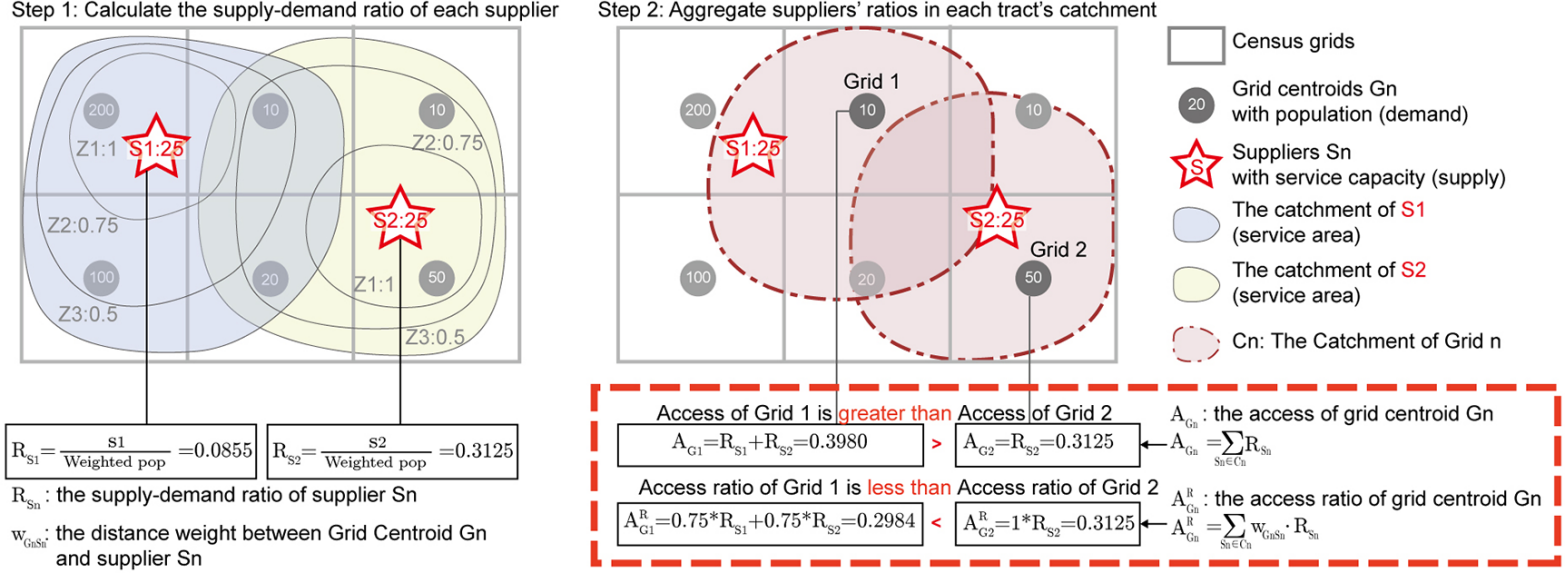
Comparison of accessibility values as the sum of supply-demand ratios ( *A*_*i*_) and the sum of weighted supply-demand ratios ( *A* ^*R*^_*i*_). *A* ^*R*^_*i*_ enlarges the resource acquisition of grids closer to supplier’s location.

Defining accessibility as the sum of weighted ratios has been widely applied in various FCA methods due to its relatively simple implementation ( Fransen et al., 2015). In the measurements of access ratios ( $A_{i}^{R}$ ), there is limited consideration of the decay coefficient value or the stability of the accessibility distribution. However, this weighted ratio has not been examined for its impact on the FCA variants. There is a potential misidentification of the ‘omnipotence’ of weights in FCA methods. For example, reused weights in population demand, which is the population multiplied by the supplier weight and the distance weight in the 3SFCA, are not equivalent to the row normalisation of the previous population (see Figure 2). The next section discusses the possible side effects of reusing weights in FCA methods.

### Side effects of reusing weights

Although researchers have discovered several limitations of FCA methods, the reuse of weights has not gained sufficient attention. Among those identified limitations (i.e. catchment sizes, overestimation of population, and the MAUP), the application of weights focuses on the value of the distance decay coefficient and the function type of distance decay ( McGrail, 2012; Bunel and Tovar, 2013). Evidently, the impacts of distance weights have been simplified to only consider the differences between decay coefficient *β* values and common forms of distance decay, including the exponential function, the inverse-power function, and the Gaussian function ( Kwan, 1998; Neutens, 2015).

However, the manipulation of weights can also have a substantial impact on modelling accessibility. Delamater (2013) has discovered one side effect of reusing weights in the 3SFCA. He modelled a simple dynamic topology that gradually moved one far-away unit closer to a facility-adjacent area. During this process, the 2SFCA method calculates the accessibility for this unit as monotonically increasing, while the 3SFCA method calculates the accessibility as first decreasing and then increasing. This is because reusing weights in population demand, which considers supplier weights and distance weights simultaneously, generates a non-monotonically varying distance decay function in the 3SFCA. Although, the non-monotonic variation in the 3SFCA may have a limited impact in complex topologies.

The use of weighted ratios can produce additional problematic results. Figure 2 illustrates one side effect of defining accessibility as the sum of weighted ratios. The grey quadrilaterals are census tracts and the red pentagrams are supplier locations. To simplify the calculation, this example applies the E2SFCA method and a piecewise function as the distance decay function. The weights of zone 1, zone 2, and zone 3 equal 1, 0.75, and 0.5, respectively. The first step calculates the supply-demand ratio of each supplier ( *S*1 and *S*2), which is the weighted population of tracts divided by the suppliers’ capacity. The second step aggregates the supply-demand ratio of suppliers within the catchment of each grid centroid. Detailed calculations are provided below:

*R*_*Sn*_: the supply-demand ratio of supplier *Sn.*

$$\begin{array}{ll} R_{S1} & =\frac{S1}{Weighted\;pop}=\frac{25}{1*200+0.75*110+0.5*20}=\frac{25}{292.5}\approx 0.0855\\ R_{S2} & =\frac{S2}{Weighted\;pop}=\frac{25}{1*50+0.75*(10+10+20)+0.5*0}=\frac{25}{80}=0.3125 \end{array}$$

*A*_*Gn*_: the access of grid *Gn* (accessibility as the sum of supple-demand ratios).

$$\begin{array}{l} A_{G1}=R_{S1}+R_{S2}=\frac{25}{292.5}+\frac{25}{80}\approx 0.3980\\ A_{G2}=R_{S2}=\frac{25}{80}=0.3125 \end{array}$$

$A_{Gn}^{R}$ : the access ratio of grid *Gn* (accessibility as the sum of weighted ratios).

$$\begin{array}{l} A_{G1}^{R}=0.75*R_{S1}+0.75*R_{S2}=0.75*\frac{25}{292.5}+0.75*\frac{25}{80}\approx 0.2984\\ A_{G2}^{R}=1*R_{S2}=0.3125 \end{array}$$

As shown in Figure 2, the access ( *A*_*i*_) of Grid 1 is greater than that of Grid 2, while their access ratios ( $A_{i}^{R}$ ) are reversed. Grid 1 has a reduced access ratio because of its location within two suppliers’ *Z*2 catchments, while Grid 2 lies within only one supplier’s *Z*1 catchment. Defining accessibility as the sum of weighted ratios may exaggerate a community’s resource consumption when it lies in close proximity to suppliers. In this instance, it remains uncertain which census tract contains higher service capacities. Still, this example reveals that access ratios exaggerate the accessibility of population units with closer proximities to suppliers and, therefore, greater geographic weights.

The exaggeration of the access ratio in units with closer proximities to amenities should be examined in the context of complicated topologies. There remains the possibility that this exaggeration is either imperative for the precise description of resource distribution, or irrelevant in the context of complex geography. Moreover, how this distortion of the access ratio relates to the modelling approach and the type of resource in question needs to be further investigated. Access ratios produced by various applications of distance weights in the FCA methods could alter its impact on accessibility results. Furthermore, due to their diverse topologies, varied resources with distinct differences in user behaviours appropriate to the size and attributes of their catchments may also influence the effect of weighted ratios. As mentioned above, there are two levels of weighting applications. The differential applications of geographical weights in supply and demand simulations shape FCA variants, while the access ratio helps quantify accessibility. Accordingly, this study focuses on the impact of the access ratio and attempts to build a systematic evaluation by comparing *A*_*i*_ and $A_{i}^{R}$ , with consideration of the aforementioned influencing factors.

## Methods

### Overview

As a global centre for finance, innovation, and transportation, Shanghai is one of the most populated metropolises in the People’s Republic of China. Its rapid urban expansion has caused a gap in distributing various resources between urban and rural areas ( Xiao et al., 2017). Accessibility measurements can detect the spatial equity of various resources in the city’s urban development. Moreover, taking Shanghai as a case study provides an empirical analysis of accessibility models in complex topology and a large city. This study chose Shanghai’s administrative land region (6340.5 *km* ^2^) as the study area.

### Data source

The original data was purchased from Urban Data Party (UDP) via its data service (please see *Underlying data*). The datasets provided by UDP are accessed from AMAP through web crawler technology. The Urban Data Party membership provides cell phone signalling data, road network data, and the points of primary schools, companies, and hospitals in Shanghai in 2017. This study used the cell phone signalling data to generate the Shanghai population grid. UDP has subsequently deleted the 2017 cell phone signalling data, and instead cooperated with WorldPop et al. (2017) to provide a broader map of population density that is publicly available.

As an alternative to the restricted UDP datasets, OpenStreetMap provides publicly available POIs and road network data of Shanghai, while WorldPop provides the population density of Shanghai (please see *Underlying data* for more information).

### Study design

This study examines the role of weighted ratios in accessibility measurements from two dimensions. The study is based on the contrast between access ( *A*_*i*_) and access ratios ( $A_{i}^{R}$ ) , as shown in Table 1. Their difference lies in whether to multiply the suppliers’ supply-demand ratios by the distance weights in the last step of the FCA methods. The first dimension is the different FCA metrics. The E2SFCA, M2SFCA, and 3SFCA models were chosen because of their prevalence and multiple weight applications with various aims. Methodological comparisons assess whether the impacts of access ratios change across different models. Therefore, the purpose of this study is to examine the relationship between the type of FCA model and the side effects of reusing geographic weights.

The second dimension is the impact of access ratios for different types of resources, including primary schools, jobs opportunities, and major hospitals. These empirical comparisons evaluate how access ratios perform in realistic and complex topology.

Table 2 shows the detailed characteristics of the three objects, including their catchment areas and spatial distributions. Primary schools are distributed dispersedly in accordance with neighbourhood locations, as pupils have limited capabilities for long-distance travel, and will therefore usually attend the school closest to their residence. In contrast, jobs and major hospitals are distributed in a centralized way, as workers and patients can typically endure longer distances to obtain income and medical services. Furthermore, healthcare-related behaviours are more tolerant to increased distance than work-related commuting behaviours because obtaining healthcare is typically a matter of necessity and occurs less frequently. Even with the same distance radius, the catchments of hospitals involve more flexible behaviours than those of job opportunities. Therefore, primary schools have a catchment radius of three kilometres for their limited-service areas, while job opportunities and major hospitals both have a 20 kilometre radius.

**Table 2.** The characteristics of three resources.

Objects	Catchment radius (km)	Number of POIs	Distance tolerance	Spatial distribution
Primary schools	3	828	Low	Dispersed
Job opportunities	20	13,488	Medium	Centralized
Major hospitals	20	277	High	Centralized

To be more specific, there are some important nuances in the tested formulae of each object in the three methods. First of all, it is crucial to realize that every tiny parameter change can lead to significant accessibility distributions, including the different sizes of catchments, different distance decay equations and different values of the distance decay coefficient *β*. However, due to different facilities usage habits, it is impossible to keep all parameters the same crossing different objects. This study tries to maintain the same parameter according to the object type. Equation (5a) and (5b) are the tested formulae of ‘access’ and ‘access ratio’ in the E2SFCA method. To avoid irrelevant influences, this study uses the Gaussian function as the distance decay equation in all three FCA methods, as Equation (5c) shows.

$$A_{i}^{E2}=\sum_{j\in C_{i}}R_{j}^{E2}=\sum_{j\in C_{i}}\frac{S_{j}}{\sum_{k\in C_{j}}w_{\mathrm{kj}}\cdot D_{k}}$$ (5a)

$$A_{i}^{R\left(E2\right)}=\sum_{j\in C_{i}}w_{\mathrm{ij}}\cdot R_{j}^{E2}=\sum_{j\in C_{i}}w_{\mathrm{kj}}\cdot \frac{S_{j}}{\sum_{k\in C_{j}}w_{\mathrm{kj}}\cdot D_{k}}$$ (5b)

$$w_{\mathrm{ij}}=\left\{\begin{array}{ll} 1 & d_{\mathrm{ij}}\;\leq \;d_{0}\\ e^{-{\left(d_{\mathrm{ij}}-d_{0}\right)}^{2}/\beta } & d_{0}<d_{\mathrm{ij}}\;\leq \;d_{c}\\ 0 & d_{\mathrm{ij}}>d_{c} \end{array}\right.$$ (5c)

$A_{i}^{E2}$ and $A_{i}^{R\left(E2\right)}$ respectively represent the ‘access’ and ‘access ratio’ calculated by the E2SFCA method, $R_{j}^{E2}$ is the supply-demand ratio of supplier *j* which falls in the catchment centred at tract *i* calculated by the E2SFCA method, *S*_*j*_ is the supplier capacity, *D*_*k*_ is the population demand of tract *k*, $w_{\mathrm{kj}}$ is the distance weight between tract *k* and supplier *j*, *d*_*kj*_ is the distance between tract *k* and supplier *j*, *β* is the decay coefficient, *d*_*c*_ is the catchment radius and *d*_0_ is the inner critical radius of the catchment, which means the tract population can be fully counted if the tract is close enough to the supplier, i.e. the distance between tract and supplier is less than *d*_0_. For jobs and major hospitals, *d*_0_ equals 2 km and their catchment radius *d*_*c*_ is 20 km. Their distances are calculated as the network distance in the driving mode. While pupils usually have low tolerance to distance, *d*_0_ for primary school is 500 m and *d*_*c*_ equals 3 km. To ensure the Gaussian equation has the same decay dimension as the power function in Equation (2b) and provides a slow changing trend at the same time ( Delamater et al., 2019), *β* equals 110 in measurements of job opportunities and major hospitals, which is 185 in measurements of primary schools.

Equation (6a) and (6b) are the ‘access’ and ‘access ratio’ calculated by the M2SFCA method. The distance decay function and coefficient remain the same as in Equation (5c).

$$A_{i}^{M2}=\sum_{j\in C_{i}}R_{j}^{M2}=\sum_{j\in C_{i}}\frac{w_{\mathrm{ij}}\cdot S_{j}}{\sum_{k\in C_{j}}w_{\mathrm{kj}}\cdot D_{k}}$$ (6a)

$$A_{i}^{R\left(M2\right)}=\sum_{j\in C_{i}}w_{\mathrm{ij}}\cdot R_{j}^{M2}=\sum_{j\in C_{i}}w_{\mathrm{ij}}\cdot \frac{w_{\mathrm{ij}}\cdot S_{j}}{\sum_{k\in C_{j}}w_{\mathrm{kj}}\cdot D_{k}}$$ (6b)

$A_{i}^{M2}$ and $A_{i}^{R\left(M2\right)}$ are the ‘access’ and ‘access ratio’ calculated by the M2SFCA method, $R_{j}^{M2}$ is the supply-demand ratio of supplier *j* which falls in the catchment centred at tract *i* calculated by the M2SFCA method, and the other variables are the same as in Equation (5a) and (5b).

Analogously, Equation (7a) and (7b) are the ‘access’ and ‘access ratio’ under the 3SFCA method. Besides the same distance decay function ( Equation (5c)), Equation (7c) is the calculation of supplier weights, which is also the main difference between *R*_*j*_ under the M2SFCA and 3SFCA methods.

$$A_{i}^{3S}=\sum_{j\in C_{i}}R_{j}^{3S}=\sum_{j\in C_{i}}\frac{S_{j}}{\sum_{k\in C_{j}}w_{\mathrm{kj}}\cdot w_{\mathrm{kj}}^{S}\cdot D_{k}}$$ (7a)

$$A_{i}^{R\left(3S\right)}=\sum_{j\in C_{i}}w_{\mathrm{ij}}\cdot R_{j}^{3S}=\sum_{j\in C_{i}}w_{\mathrm{ij}}\cdot \frac{S_{j}}{\sum_{k\in C_{j}}w_{\mathrm{kj}}\cdot w_{\mathrm{kj}}^{S}\cdot D_{k}}$$ (7b)

$$w_{\mathrm{kj}}^{S}=\frac{w_{\mathrm{kj}}}{\sum_{m\in C_{k}}w_{\mathrm{km}}}$$ (7c)

$A_{i}^{3S}$ and $A_{i}^{R\left(3S\right)}$ are the ‘access’ and ‘access ratio’ calculated by the 3SFCA method, $R_{j}^{3S}$ is the supply-demand ratio of supplier *j* which falls in the catchment centred at tract *i* calculated by the 3SFCA method, $w_{\mathrm{kj}}^{S}$ is the supplier weight between tract *k* and supplier *j*, where supplier *m* is the supplier's fall in the catchment of tract *k*, and *w*_*km*_ is the distance weights between tract *k* and supplier *m*, and the other variables are the same as in Equation (5a) and (5b).

### Data analysis

There are three stages of data analysis: data pre-processing, generation of the distance matrices and the calculation stage. The first pre-processing stage provides the basic datasets for the distance matrices. The distance matrices refer to the network distance between each population grid and each POI. The calculation stage computes the values of access and access ratios for each grid.

The first stage processes the population grid and the POI datasets in the software Quantum Geographic Information System ( QGIS 3.16.6). This study first generated the population density from the 2017 cell phone signalling data for the population grid. It then used the default Zonal Statistics plug-in in QGIS to transfer the population density into a 250*250 *m* population grid (28,037 units) in conjunction with the Statistical Yearbook data ( Shanghai Statistics Bureau, 2018). For the POIs downloaded from UDP (or OpenStreetMap), it is necessary to delete wrong and duplicate points in the QGIS. Specifically, the number of companies (over two hundred thousands points) is too large to generate distance metrics in QGIS. Therefore, the job opportunities are estimated in the 250*250 *m* grid by multiplying the company size and the number of companies. Major hospitals are those whose official level is greater than Grade II. All service capacities are calculated as the number of objects. For datasets downloaded from OpenStreetMap and WorldPop, it is crucial to ensure they have the same coordinate systems and projections.

The second stage is to form the distance matrices between population grids and POIs datasets. The QNEAT3 plug-in in QGIS generated the distance matrices between the population grid’s geometric centre points and the POIs based on the road networks. In this study, the distance is computed in the car-drive mode.

Based on the distance matrices, the third stage calculates the spatial accessibilities to three types of facilities within three FCA models. Python (3.7.0) is used to calculate the accessibility of each population grid in three existing models (2SFCA, M2SFCA, and 3SFCA). Using the custom codes available in *Extended data* ( Zhang, 2021) can realise the calculations of access ( *A*_*i*_) and access ratios ( $A_{i}^{R}$ ) in the three models. An alternative is to reference the codes of access ( *A*_*i*_) in 2SFCA and 3SFCA models at the spatial_access package. This package requires strict forms of original data and generates limited accessibility results.

## Results

**Figure 3.**
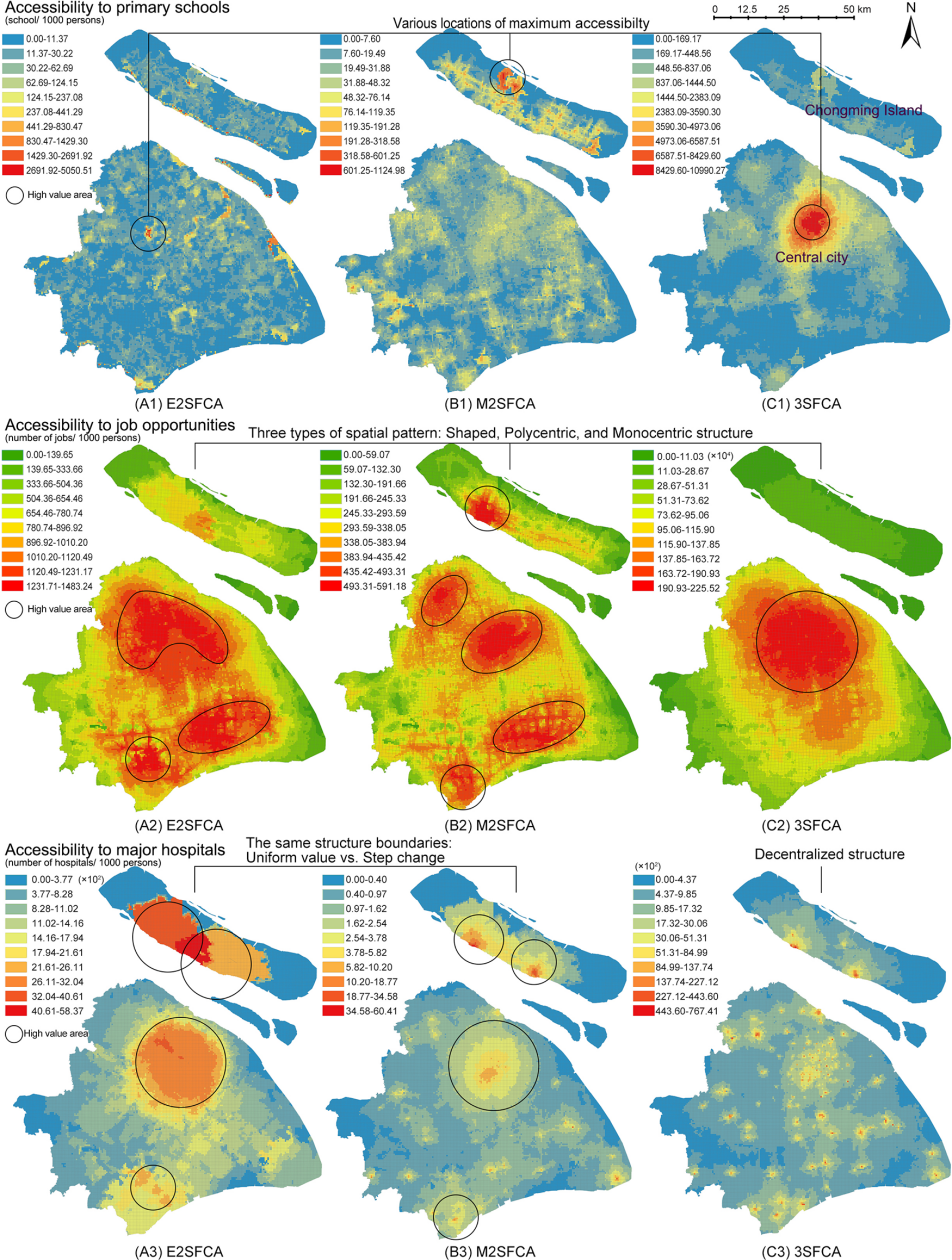
Access as the sum of supply-demand ratios ( *A*_*i*_). Three floating catchement area (FCA) metrics generate significantly different spatial structures of accessibility. (A) enhanced two-step FCA (E2SFCA), (B) modified two-step FCA (M2SFCA), and (C) three-step FCA (3SFCA).

**Figure 4.**
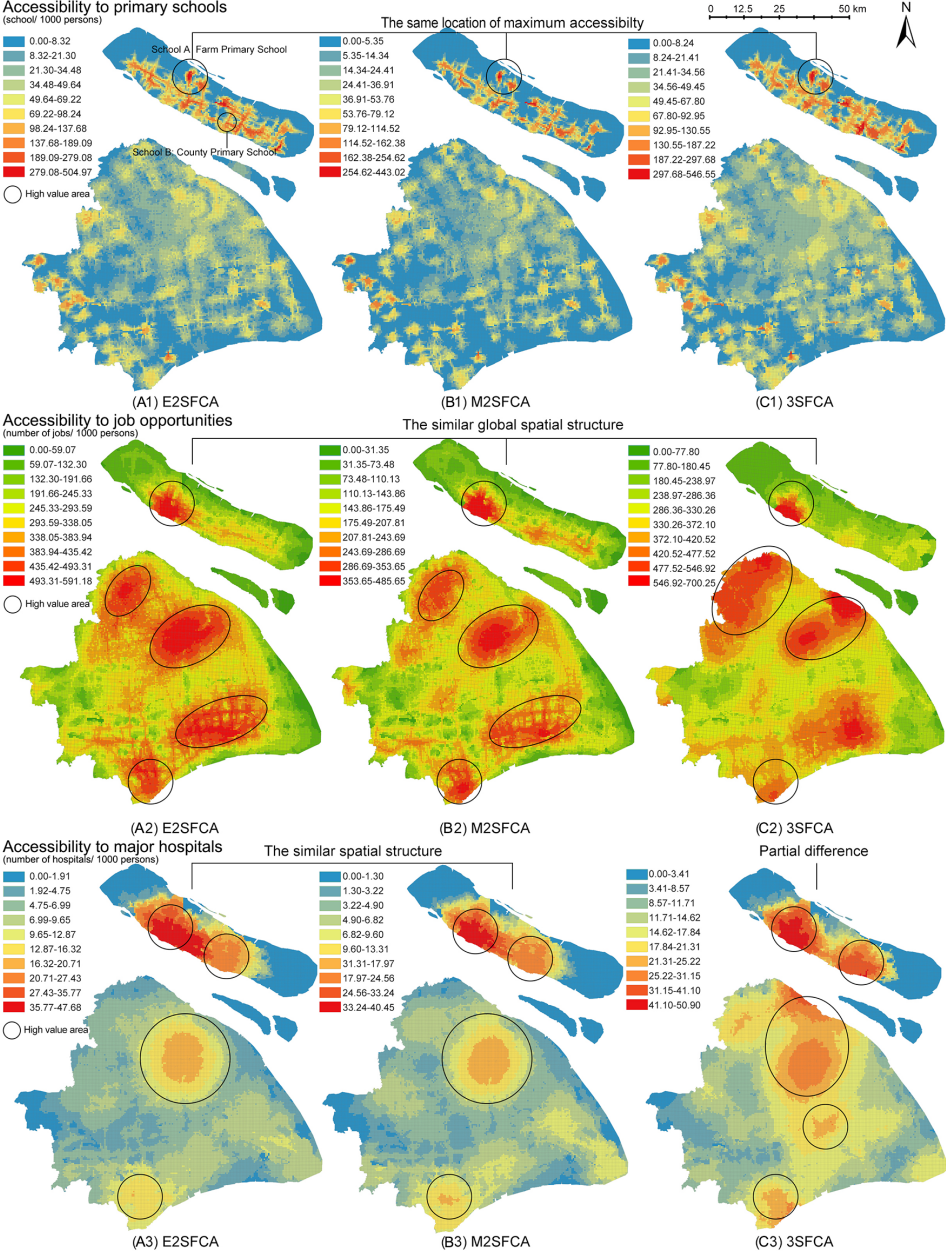
Access ratios as the sum of weighted supply-demand ratios ( *A* ^*R*^_*i*_). Three floating catchement area (FCA) metrics generate similar spatial distribution of accessibility according to resources. (A) enhanced two-step FCA (E2SFCA), (B) modified two-step FCA (M2SFCA), and (C) three-step FCA (3SFCA).

As a continuation of the previous study design outlined in the Methods, the analysis examines methodological and empirical implications of the results by comparison of *A*_*i*_ and $A_{i}^{R}$ . Figure 3 presents the access results ( *A*_*i*_), meanwhile, Figure 4 presents the results of access ratios ( $A_{i}^{R}$ ). In Figure 3, the three FCA metrics generate significantly different spatial structures, whereas Figure 4 shows similar structure of access ratios for the resources.

### Weighted ratios: Pattern stability beyond model types

From a methodological perspective, weighted ratios produce stable accessibility patterns regardless of the FCA model type. Compared to the non-weighted maps ( Figure 3), weighted ratios ( Figure 4) result in similar global spatial structures of accessibility distribution. The weighted ratios produce stable patterns not only under different values of the distance decay coefficient ( Wan et al., 2012a), but also under various FCA methods. Particularly for resources with a low distance tolerance, such as primary schools, weighted ratios produce maximum accessibility to the same locations. As the tolerance of user behaviour increases, the nuances between the three methods become more pronounced. The 3SFCA creates more local differences in global structure between job accessibility and health accessibility compared to E2SFCA and M2SFCA. This implies that user behaviours in resource utilization and the aggregation of original POIs can affect the stability of the weighted ratios.

One possible explanation for this pattern stability is the side effect of weighted ratios shown in Figure 2. Weighted ratios overestimate the accessibility to resources of communities with high distance weights and a close proximity to suppliers, as higher access ratios are observed in peripheral areas rather than city centres (see Figure 4). Units in peripheral areas tend to have fewer suppliers in their catchments, resulting in high supplier weights. For those units with only one facility and close proximity to it, their access ratios ( $A_{i}^{R}$ ) are almost the same as their access values ( *A*_*i*_). Conversely, units in the city centre with multiple suppliers have access ratios lower than their access values because of their relative small distance weights multiplied several times. Therefore, access ratios of units with fewer suppliers in the periphery are relatively enlarged.

However, there remains a common point between weighted and non-weighted ratios. Both results show that the accessibility distribution differs depending on the type of amenity. It is logical that accessibility structures for primary schools, jobs, and main hospitals are dissimilar, as each object has its own distinguishing distribution. This suggests that the accessibility maps have an evidence-based foundation in addition to methodological influences.

Although weighted ratios eliminate the effect of the decay coefficient values, the validity of model stability across model types remains contentious. In Figure 4, the nuances between three FCA methods exist in partial districts and at the local level. Those subtle differences make it difficult to verify the intended characteristics of FCA methods and further lead to the dispute over the necessity of improvements. On the contrary, Figure 3 visualises the different model improvements in results. The improvement of the M2SFCA, that is, the distance decay of service effectiveness, converts the uniform value of accessibility into a gradual value (especially A3 and B3 in Figure 3). The enhancement of the 3SFCA, which models demand division among suppliers, reduces the overestimation of the population in the facility cluster and produces a more concentrated distribution (see B1, C1, B2 and C2 in Figure 3). On the other hand, it could also be considered a methodological advantage of weighted ratios to create stability across FCA methods. To determine which calculation, using either non-weighted or weighted ratios, is rational, one possible approach is to apply the evidence-based foundation and introduce empirical judgements.

### An epistemological trap

Although their results vary, both calculations of access and access ratios are supported by methodological rubrics. Despite these methodological rationalisations, the variability of results leads to diametrically opposed empirical judgements. These contrasting discernments could make it easier to choose a more rational method, based on experiences. Therefore, the evidence-based foundation of accessibility measures may also provide empirical criteria for the choice between *A*_*i*_ and $A_{i}^{R}$ , by linking the mapped results with common sense.

While there are varying degrees of variability between access and access ratios for different resources, they show a common characteristic among the resources. Maps of access ratios ( $A_{i}^{R}$ in Figure 4) share similar structures with those of the access calculated by the M2FCA ( *A*_*i*_ B-maps in Figure 3). This is due to the reuse of weights in the M2SFCA, as described in the formulas shown in Table 1. Furthermore, the reused weights divide accessibility distribution into two kinds of judgements. In the case of primary schools, the access ratios in Figure 4 indicate that the central city contains a shortage of primary education resources, while the north-eastern island (Chongming Island) has the greatest abundance of those resources. However, the 3SFCA method in Figure 3 concludes the opposite: that the city centre is the most resource-rich, while Chongming Island lacks basic education facilities. Similarly opposed conclusions are the polycentric and monocentric structures of accessibility to job opportunities and major hospitals generated from *A*_*i*_ and $A_{i}^{R}$ .

The difficulty in judging the validity of opposing results lies in the ratio attribute of accessibility. The provision of services and the needs of the population are reflected in visual and definitive indicators of their quantity. Each facility points has certain indicator of its service capacity, such as the number of physicians in a hospital. Each residential unit obtains an accurate number for its population. However, the relationship between supply and demand is indirect, uncertain, and dynamic. The maximum supply-demand ratio could occur in the suburbs or the city centre based on empiricism.

One possible solution is to apply Kantian ‘ *a priori* knowledge’, which combines empiricism and rationalism. First, key locations are contrasted to provide actual and intuitive measurements. Field research on the key locations provide empirical evidences for the resource distribution. Second, the overall judgements of resource distributions in previous studies are discussed to choose the most accurate global structure of accessibility. These previous studies provide the ‘ *a priori* knowledge’ of resource distributions based on actual utilizations.

Fieldwork may provide insight into the comparison of the accessibility values of key points and thus inform model recommendations. In accessibility to primary schools, the catchment of School A at Chongming Island (see (a1) in Figure 4) has one of the highest access ratios $A_{i}^{R}$ , although the island is famous for its agricultural products and natural landscapes. School B is one of the medium values also located on the island. Field research reveals that School A serves only its collective farm, which is a socialist legacy with limited productivity and a decreasing population. School B, on the other hand, is located in a more densely populated area and provides a higher level of educational resources for the children in the county. It is counter-intuitive that the catchment area of School A is more accessible than School B.

Furthermore, the urban-rural dichotomy regarding quality of life caused by the Chinese dual system has been a long-standing issue ( Chan and Wei, 2019; Ma et al., 2020). In the dichotomy, population and land management systems are both separate and unequal between urban and rural areas. The socialist governance system leads to the concentration of public resources in the urban areas, while rural areas lack the same resources. Xiao et al. (2017) identified that high access to urban parks occurred mainly in downtown Shanghai. Zhao and Cao (2020) investigated 81 million trips that used transit smart cards to identify the gradual decrease of job accessibility from the city centre towards the outer suburbs. In the case of hospitals, the urban-rural dichotomy of medical resources has already been identified, revealing a lack of medical resources in suburbs ( Meng et al., 2015). In 2009, the Shanghai Municipal Government launched the ‘5+3+1’ project to promote the construction of high-quality hospitals in the periphery. Using this ‘ *a priori* knowledge’, it seems impossible that the most abundant resources are located in the agricultural island in all three categories. Therefore, the 3SFCA model with non-weighted ratios might be the most appropriate method to reflect the accessibility distribution in Shanghai.

In summary, the reuse of weights, including the M2SFCA method ( Figure 3 b-maps) and defining accessibility as the sum of weighted ratios ( Figure 4), can generate an epistemological trap. The outcomes of access ratios can produce pattern stability incongruous with model features and resource types, when compared to the access results. Moreover, the side effect of reusing weights, which exaggerates accessibility for units with closer proximities and fewer suppliers, can produce counter-intuitive results. The reuse of weights may lead to methodologically plausible, but common-sense-defying distributions of accessibility. It is therefore necessary to combine empiricism and rationalism in the evaluation of accessibility methods; similarly, the use of geographic weights needs to be cautious and epistemologically consistent.

## Discussion and conclusions

Spatial equity and distributive justice have been controversial issues due to their elusive concepts and complex assessments ( Guagliardo, 2004; Hay, 1995). Identifying spatial inequity requires recognising the areas with shortages of social resources. Accessibility, as a fundamental indicator of spatial equity, assesses the distribution of urban resources by integrating supply-demand ratios and distance decay into FCA metrics. However, varying parameters and flexible applications of FCA metrics could generate methodologically logistical but empirically counter-intuitive results.

This study reveals that the reuse of weights might lead to an epistemological trap and misperceptions of resource distribution. The reuse of weights in FCA metrics, which is the multiple use of weights in simulating the decay of supply capacity, population demand, and the supply-demand ratios, may exaggerate accessibility in marginal communities that are within fewer suppliers’ catchments. Whereas past researchers have found that catchment size should be set with caution ( McGrail and Humphreys, 2009), the present study has shown the use of distance weights may also require vigilance. Comparing the results of access (the sum of supply-demand ratios) and access ratios (the sum of weighted supply-demand ratios) across three different resources shows that weighted ratios produce pattern stability beyond the model features of the E2SFCA, M2SFCA, and 3SFCA methods. On the contrary, non-weighted ratios may help to visualize the model characteristics most accurately. Linking the model results with empirical judgements, this study suggests that the 3SFCA method using non-weighted ratios can achieve the most realistic accessibility distribution among three resources in Shanghai.

There are at least three potential limitations concerning the results of this study. First, the results are limited by the city-level spatial scale and the topographic features of Shanghai. Smaller spatial scale and less complex environments could alleviate the exaggeration of weighted ratios. Shanghai’s polycentric city structure, as well as its islands, might also contribute to the problematic dispersed resource distributions. Further, the assessment is based on the local-level POI data and contains numerous supplier points, which can cause methodological fallacies in this specific combination of resource distribution, city structure, and unit aggregation. Fewer suppliers, even with divergent catchment areas, still generate simple topologies and may not encounter the trap of weights ( Pan et al., 2018). Lastly, this study still produces place-based assessments with the FCA metrics, which lacks detail of the individual differences in facility utilization. Further studies can explore the solutions for identifying individual preferences in facility utilization and dealing with personal uncertainties.

Despite these limitations, this study provides insights for a better understanding of accessibility and GIS modelling. For accessibility measurements, this examination of reused weights in FCA metrics raises the issue of evaluation criteria for improving methods. The socioeconomic attributes of accessibility measurement include social and cultural factors in the use of facilities and the organisation of resources. This socioeconomic attribute requires assessing improvements that have not only a methodologically logical necessity, but also a practical implication. This study reveals that sensible logic for the application of distance weights needs further exploration. Since the first law of geography argues that near things are more related than distant things ( Tobler, 1970), the applications of distance weights have been a crucial method to represent this relationship. The study will prove useful in expanding our understanding of how distance weights should be applied in GIS models. Last but not least, the present study distinguishes the gap between methodological rational and empirical judgements in GIS modelling. It is difficult to discern whether the results of GIS models generated by rational techniques reflect the real situation. Understanding this reality is a matter of epistemology. Methodological fallacies, such as the trap of weights, need to be examined with empiricism and rationalism in further studies.

## Data availability

### Underlying data

The underlying data consists of the cell phone signalling data, road networks, point of interest (POI) data (primary schools, companies, and hospitals) in Shanghai in 2017, which was purchased from Urban Data Party (UDP) via its data service. These datasets cannot be made publicly available because they are under copyright to Urban Data Party. The following URLs are only available with Urban Data Party member registration:

• Points of education facilities: https://www.udparty.com/index.php/detail/articledetails/?id=4502…title=19\%E5\%B9\%B4\%E4\%B8\%8A\%E6\%B5\%B7\%E5\%B8\%82\%E6\%95\%99\%E8\%82\%B2\%E5\%A4\%A7\%E7\%B1\%BBPOI\%E6\%95\%B0\%E6\%8D\%AE
• Points of companies and hospitals: https://www.udparty.com/index.php/detail/articledetails/?id=1585…title=\%E4\%B8\%8A\%E6\%B5\%B7\%E5\%90\%84\%E7\%B1\%BBPOI\%E6\%95\%B0\%E6\%8D\%AE\%E6\%B1\%87\%E6\%80\%BB
• Shanghai road network: https://www.udparty.com/index.php/detail/articledetails/?id=3820…title=\%E4\%B8\%8A\%E6\%B5\%B7\%E5\%B8\%82\%E9\%81\%93\%E8\%B7\%AF\%E6\%95\%B0\%E6\%8D\%AE\%EF\%BC\%882018\%E5\%B9\%B411\%E6\%9C\%88\%EF\%BC\%89
• Population density: Urban Data Party has since deleted the cell phone signalling data and updated the population density from WorldPop: https://dx.doi.org/10.5258/SOTON/WP00675.

As an alternative to the restricted UDP datasets (points 1-3), OpenStreetMap provides publicly available POI and road network data for Shanghai which is representative of the analysed datasets: https://www.openstreetmap.org/relation/913067. This data can be downloaded directly on the website via the Overpass API. The QGIS plug-in QuickOSM can also download the OpenStreetMap data and convert it to shapefiles, see tutorials here.

### Extended data

Zenodo: Python codes for accessibility and access ratios in the E2SFCA, M2SFCA, 3SFCA methods. http://doi.org/10.5281/zenodo.4890880 ( Zhang, 2021).

This project contains the following extended data:

• 01-E2SFCA.py
• 02-M2SFCA.py
• 03-3SFCA.py
• LICENSE.txt
• readme.txt

Data are available under the terms of the Apache License 2.0.

## Author endorsement

Prof. Dr. Frank Othengrafen confirms that the author has an appropriate level of expertise to conduct this research, and confirms that the submission is of an acceptable scientific standard. Prof. Dr. Othengrafen declares they have no competing interests. Affiliation: TU Dortmund University, Dortmund, 44149, Germany.
