# Supplementary material for: Trap of weights: The reuse of weights in the floating catchment area (FCA) methods to measuring accessibility
Source: F1000Res. 2022 Feb 8;10:751. Originally published 2021 Aug 4. [Version 2] doi: 10.12688/f1000research.51483.2 (PMC10130702; doi:10.12688/f1000research.51483.2)
Supplement: Peer review history

# Peer review: Reviewer response for version 2

**DOI:** 10.5256/f1000research.120179.r167486

**Published:** 2023-04-25

**Authors:** Fahui Wang

**Affiliations:** 1 Department of Geography and Anthropology, Louisiana State University, Baton Rouge, Louisiana, USA

The paper uses 3 case studies in Shanghai to implement the popular 2SFCA method, now framed in a new term "spatial weights," and examines the impact/sensitivity of various ways of defining spatial weights.

 My assessment is that the conceptualization of distance decay functions in "spatial weights" may be considered an innovation. In other words, the variants of 2SFCA method differ in how the supply and demand are weighted in response to distance (or spatial relationship). As early as 2012, Wang (Annals of AAG 102: 1104-1112 ^1^) integrated the family of 2SFCA models into one framework termed “Generalized 2SFCA method”, where various models believe/choose a different distance decay function to rescale both supply and demand. Here, Zhang uses a term “spatial weights” for the same concept. I am surprised that Wang (2012) ^1^ is not cited here.

 It is a good exercise for the author to examine the differences in various “spatial weights” via multiple applications (school, jobs, hospitals), and caution people to take their results with a grain of salt. The key unanswered question is: what spatial weights are the best? Is there a way to find them? The answer is offered in Wang (2012, pp.1107 - ^1^): “Any debate over the best function or the right size for catchment areas cannot be settled without analyzing real-world health care utilization behavior.”

 Moreover, there has been some significant development in recent work related to 2SFCA. For example, Wang (2021, IJGIS 35: 628-638 - ^2^) provided theoretical proof to the model, and thus in a way to reject the need of added complexity of 3SFCA etc. That needs to be noted.

Is the work clearly and accurately presented and does it cite the current literature?

Partly

If applicable, is the statistical analysis and its interpretation appropriate?

Yes

Are all the source data underlying the results available to ensure full reproducibility?

Yes

Is the study design appropriate and is the work technically sound?

Yes

Are the conclusions drawn adequately supported by the results?

Yes

Are sufficient details of methods and analysis provided to allow replication by others?

Yes

Reviewer Expertise:

GIS and spatial computational social sciences

I confirm that I have read this submission and believe that I have an appropriate level of expertise to confirm that it is of an acceptable scientific standard.

## References

[1] : Measurement, Optimization, and Impact of Health Care Accessibility: A Methodological Review. *Ann Assoc Am Geogr* .2012;102(5) : 10.1080/00045608.2012.657146 1104-1112 10.1080/00045608.2012.657146 23335813PMC3547595

[2] : From 2SFCA to i2SFCA: integration, derivation and validation. *Int J Geogr Inf Sci* .2021;35(3) : 10.1080/13658816.2020.1811868 628-638 10.1080/13658816.2020.1811868 33732091PMC7959250

# Peer review: Reviewer response for version 2

**DOI:** 10.5256/f1000research.120179.r122784

**Published:** 2022-03-25

**Authors:** Matthew R. McGrail

**Affiliations:** 1 Rural Clinical School, University of Queensland, Qld, Australia

No further comments, thanks for addressing my concerns.

Is the work clearly and accurately presented and does it cite the current literature?

Partly

If applicable, is the statistical analysis and its interpretation appropriate?

Yes

Are all the source data underlying the results available to ensure full reproducibility?

Yes

Is the study design appropriate and is the work technically sound?

Yes

Are the conclusions drawn adequately supported by the results?

Partly

Are sufficient details of methods and analysis provided to allow replication by others?

No

Reviewer Expertise:

Health services research - workforce distribution & access to health care

I confirm that I have read this submission and believe that I have an appropriate level of expertise to confirm that it is of an acceptable scientific standard.

# Peer review: Reviewer response for version 1

**DOI:** 10.5256/f1000research.54657.r99589

**Published:** 2021-11-22

**Authors:** Matthew R. McGrail

**Affiliations:** 1 Rural Clinical School, University of Queensland, Qld, Australia

I congratulate the author for conducting this study. I agree that amongst the growth in use and testing of the broad family of FCA methods, it remains that catchment size and distance weights are under-researched. Some of my own research focused more so on catchment sizes; this study here focuses on the way that distance weights are applied.

 I’m confident that what the authors have tested and tried to present here is a significant addition to the evidence base. However, I’m still not 100% sure exactly what specifically the authors have actually tested – my expectation is that it will require minor clarification to address.

 The authors specifically state early (in the Intro) that “for clarity, this study defines the original non-weighted accessibility as ‘access’ and the weighted ratios as ‘access ratios’. I have a high-level understanding the broad 2SFCA family of methods, but my confusion is tied to this statement (and thus how it is applied in the paper). Despite what Table 1 presents, I have not been able to understand what the Access (Ai) scores exactly relate to.

- Is non-weighted ‘access’ for the E2sfca equivalent to the original 2sfca (i.e. without weights), or something else?
- In Table 1, the M2sfca Ai formula includes a weight, thus how is this non-weighted?
- I do not understand the difference between the Ai formula for E2sfca vs 3sfca? As per Figure 3, they clearly produce different answers but I can’ understand what specifically is being calculated differently – Table 1 suggests otherwise.
- Clarification of specifically what the authors have done (e.g. relate then back to the equations (1) to (4)) will help enormously.

Similarly, am I to assume that the right column of Table 1 equivalent to the original 3 methods (written in a ‘short-hand’ way (like Figure 1), or have they applied some other modification?

- Again, clarification of this ‘simple’ point will help enormously.

This misunderstanding aside, I really like what they’ve done in Figures 3 and 4.  The results are presented and explained very clearly (even if I haven’t quite understood the underlying methods being compared). I also like the comparisons of the 3 types of resources. This gives the reader a broader understanding of the outcomes seen in Figs 3 and 4 and how each method relates to the different characteristics as per Table 2.

 I did not find Figure 2 helpful, it was quite confusing trying to understand specifically what it is trying to demonstrate at that point (it did not help me interpret Table 1, which is critical to the paper).

 Apart from clarifying data sources and their GIS processes, the Methods section gives little detail on the specific formulae being tested (simply pointing back to Table 1). This is the critical information missing in the paper. As an aside, it is highly unusual for the Methods section to point the reader BACK to the Intro / Background sections for the detail.

 Just before the Discussion & Conclusion section, I really like their statement of “the reuse of weights may lead to methodologically plausible but common-sense defying distribution of accessibility”; however as per above, I think the reader is currently lacking some specific technical information of the methods to 100% appreciate this study’s evidence.

Is the work clearly and accurately presented and does it cite the current literature?

Partly

If applicable, is the statistical analysis and its interpretation appropriate?

Yes

Are all the source data underlying the results available to ensure full reproducibility?

Yes

Is the study design appropriate and is the work technically sound?

Yes

Are the conclusions drawn adequately supported by the results?

Partly

Are sufficient details of methods and analysis provided to allow replication by others?

No

Reviewer Expertise:

Health services research - workforce distribution & access to health care

I confirm that I have read this submission and believe that I have an appropriate level of expertise to confirm that it is of an acceptable scientific standard, however I have significant reservations, as outlined above.

# Response

**Published:** 2021-12-13

**Authors:** Lina Zhang

**Affiliations:** TU Dortmund University, Germany

The author would like to thank the reviewer for his kind comments and valuable questions. I have carefully read the referee's report and will revise the manuscript according to the referees' suggestions after the second reviewer’s report. I also feel quite sorry for the unclear articulation of the key concept in this first vision.  It seems to be quite necessary to answer some questions mentioned in the report in advance.

- * “The authors specifically state early (in the Intro) that “for clarity, this study defines the original non-weighted accessibility as ‘access’ and the weighted ratios as ‘access ratios. I have a high-level understanding the broad 2SFCA family of methods, but my confusion is tied to this statement (and thus how it is applied in the paper).”*
- **Response:**
  - This statement should be revised in the Intro. While distance weight has various applications in FCA methods, this study focuses on its application **in the final step** of FCA methods. The difference between ‘weighted’ and ‘non-weight’ refers to the second step in the 2sfca and the third step in the 3sfca method, which calculates the accessibility of one community to facilities.
  -  If we focus on the original 2SFCA method, then the first step is to calculate the supply-demand ratio of certain facility *Rj *and the second step is to calculate the sum of supply-demand ratio of certain unit *Ai*. For the second step, the very first method (2sfca) does not include distance weight ( ) (Luo & Wang, 2003).  However, there is another vision of this step in the enhanced-2sfca method, which multiplies the facility supply-demand ratio by distance weight  ( ) (Luo & Qi, 2009). The former one is the original non-weighted accessibility ‘access’, while the latter one is the ‘access ratio’.
  - In the case of the 3sfca method, if we regard the first two steps (step 0: the calculation of supplier weight and step1: the calculation of *Rj*) as one finer step of *Rj* calculation, then the problem remains quite similar in the E2sfca method. The key question here is whether we should multiply *Rj* by distance weight and supplier weight in the final step (  vs.  ).
  - In the case of M2SFCA, I made a quite important mistake because of the complicated situation. And I believe this mistake raises major confusion. In Table 1, the M2sfca Ai formula is inaccurate. It should be . This mistake is made because I would emphasise the different calculation processes. In the previous two methods (E2sfca and 3sfca), the supply-demand ratio *Rj* belongs to one facility *j*. While in the M2sfca, the supply-demand ratio is calculated for each pair of facility *j* and population unit *i*. Because Delamater (2013) constructs a specific supply ratio for each facility *j* and unit *i* in the first step. To reflect this difference in the calculation process, I emphasized the *w _ij _* in the first step of M2sfca in Table 1, which is unnecessary.  However, the second step in the M2sfca is the same as the E2SFCA. So, the key question remains the same, that is, whether we should multiply *Rj *by the distance weight in the second step of the M2sfca. Therefore, the ‘access’ in M2sfca should be . And ‘access ratio’ should be .
- *“Despite what Table 1 presents, I have not been able to understand what the Access (Ai) scores exactly relate to. Is non-weighted ‘access’ for the E2sfca equivalent to the original 2sfca (i.e. without weights), or something else? ”*
- **Response: **No, the non-weighted ‘access’ for the E2sfca is not equivalent to the original 2sfca. Their difference lies in the first step, which is the calculation of supply-demand ratio Rj of certain facility *j*. In the original 2sfca, *Rj *is the supplier capacity divided by the population. In the E2sfca, Rj is the supplier capacity divided by the weighted population. The ‘non-weighted access’ for the E2sfca is the sum-up of** . And the ‘weighted access ratio’ for the E2sfca is the sum-up of .
- *“In Table 1, the M2sfca Ai formula includes a weight, thus how is this non-weighted?”*
- **Response: **The M2sfca Ai formula is inaccurate. It should be** The M2sfca *Ai* indeed includes distance weight, which is used to calculate the service supply ratio *Rj *in the first step. While this study focuses on the weight in the second step. The non-weighted ‘access’ in the second step refers to the access which sums up the *Rij* of each facility and each community without distance weight. On the contrary, the weighted ‘access ratio’ is to multiply each *Rij* by their distance weights *wij*.
- *“I do not understand the difference between the Ai formula for E2sfca vs 3sfca? As per Figure 3, they clearly produce different answers but I can’ understand what specifically is being calculated differently – Table 1 suggests otherwise.”*
- **Response: **I guess I should add one more paragraph to explain the difference between the Ai formula for E2sfca and 3sfca. The difference between the *Ai* formula for E2sfca and 3sfca lies in the calculation of facilities’ supply-demand ratio Rj. To be more precise, they differ mainly in the way they calculate the population demand. In E2sfca, the population demand of certain facility is the units’ population multiplied by distance weight. However, Wan et al. (2012) find this method is quite problematic. Because the 'unit' population is double-counted as the number of facilities increases. For example, if one unit has three facilities in its catchment, then the unit’s population is calculated three times. Each facility’s supply-demand ratio includes its population as demand.  If the number of facilities in the unit’s catchment increases to ten, then this unit’s population is calculated ten times. This double-counted population causes enlarged demand in facility concentration areas and thus decreases the accessibility in those areas. To solve this problem, the 3sfca introduces one more step before the original Rj calculation, which divides the unit’s demand according to each pairwise weight of facility and unit. In 3sfca, the population demand of certain facility is the units’ population multiplied by distance weight and supplier weight.
- Similarly, am I to assume that the right column of Table 1 equivalent to the original 3 methods (written in a ‘short-hand’ way (like Figure 1), or have they applied some other modification?
- **Response: **
  - Yes, the right column of Table 1 is equivalent to the original 3 methods. I guess this simple answer might remain some confusion on methodological understanding. So further details are provided as follow:
  - If we divide the use of weights in FCA into two categories: one that applied in the calculation of facilities’ supply-demand ratio *Rj*, and the other applied in the calculation of accessibility, then the answer for this question might be clearer. The difference between non-weighted ‘access’ and weighted ‘access ratio’ is whether to multiply *Rj* by the distance weight, that is, the final step of FCA methods. The difference between methods (E2sfca, M2sfca, and 3sfca) is how to calculate *Rj*, that is, the first step of 2sfca methods and the first two steps of 3sfca.
  - I hope the above explanation could clarify the key comparison (Ai vs. Ar) of this study. Further modifications to Figure 2 and the specific formulae in the Methods section's will be placed in the next version.

**References:**

- Delamater, P. L. (2013). Spatial accessibility in suboptimally configured health care systems: a modified two-step floating catchment area (M2SFCA) metric. *Health \& Place*, *24*, 30-43. https://doi.org/10.1016/j.healthplace.2013.07.012  
- Luo, W., & Qi, Y. (2009). An enhanced two-step floating catchment area (E2SFCA) method for measuring spatial accessibility to primary care physicians. *Health \& Place*, *15*(4), 1100-1107. https://doi.org/10.1016/j.healthplace.2009.06.002  
- Luo, W., & Wang, F. (2003). Measures of spatial accessibility to health care in a GIS environment: synthesis and a case study in the Chicago Region. *Environment and Planning B: Planning and Design*, *30*(6), 865-884. https://doi.org/10.1068/b29120               
- Wan, N., Zou, B., & Sternberg, T. (2012). A three-step floating catchment area method for analyzing spatial access to health services. *International Journal of Geographical Information Science*, *26*(6), 1073-1089. https://doi.org/10.1080/13658816.2011.624987             

# Response

**Published:** 2022-01-11

**Authors:** Lina Zhang

**Affiliations:** TU Dortmund University, Germany

This response is an additional explanation of the reviewer's report and provides further details of the second vision of the manuscript. First of all, the author would like to appreciate the referee for his thoughtful remarks and valuable suggestions. I revised the manuscript according to the referee’s suggestion. Some problems left in the previous response are answered as follows.

- *The definitions of ‘access’ and ‘access ratios’ are not clear, including the statement in the Introduction and the formulas in Table 1.“The authors specifically state early (in the Intro) that “for clarity, this study defines the original non-weighted accessibility as ‘access’ and the weighted ratios as ‘access ratios’. I have a high-level understanding of the broad 2SFCA family of methods, but my confusion is tied to this statement (and thus how it is applied in the paper). Despite what Table 1 presents, I have not been able to understand what the Access (Ai) scores exactly relate to. ”*
- **Response:**
  - This statement in the Introduction has been revised. A detailed explanation has been added to the penultimate paragraph. And the difference between ‘weighted’ and ‘non-weight’, i.e. whether distance weights are applied in the last step of the FCA methods, has been highlighted in the first sentence of the last paragraph in the Introduction.
  - The formulas and footnotes in Table 1 have been corrected and revised. The M2sfca Ai formula in Table 1 was inaccurate, which should not include weight. The explanation of the difference between the Ai formula for E2sfca, M2sfca, and 3sfca follows the Table 1 illustration, as shown below.
  - “Table 1 shows the comparison of access Ai and access ratio Ar of the three methods. The difference between 'access' and 'access ratio' is whether to multiply the suppliers' supply-demand ratios (Rj) by distance weights (wij) in the last step. The differences between E2SFCA, M2SFCA, and 3SFCA is the way to calculate the suppliers' supply-demand ratios (Rj). The E2SFCA calculates Rj as supplier capacity divided by the weighted population. The M2SFCA argues that the supplier capacity also decreases while distance increases and multiplies supplier capacity by the distance weight. The 3SFCA reveals the double-counted population demand and multiplies population demand by the distance weight and the supplier weight.”
- *Figure 2 provides limited information on the difference between ‘access’ and ‘access ratios’. “I did not find Figure 2 helpful, it was quite confusing trying to understand specifically what it is trying to demonstrate at that point (it did not help me interpret Table 1, which is critical to the paper).”*
- **Response: **The equations of ‘access’ and ‘access ratio’ has been added in Figure 2. I also added a red dotted box to clarify the focus of Figure 2. In the same distribution of supplier and census grid, one grid’s access might be greater than the other, while whose access ratio is less than the other’s access ratios. It shows that different calculations of accessibility (the ‘access’ and ‘access ratio’ in this case) might lead to magnificent changes in accessibility distributions.
- “ *Apart from clarifying data sources and their GIS processes, the Methods section gives little detail on the specific formulae being tested (simply pointing back to Table 1). This is the critical information missing in the paper.”*
- **Response: **I added the specific formulae being tested in the Study Design section of the Methods section. Besides, necessary parameters settings of the three FCA methods and three objects have also been added.  
